# Simultaneous Volumetric T1 and T2 Mapping With Blood‐ and Fat‐Suppression in Abdominal Aortic Aneurysms

**DOI:** 10.1002/mrm.70297

**Published:** 2026-02-10

**Authors:** Wilhelm Stehling, Myrte Wennen, Eva Aalbregt, Eric Schrauben, Pim van Ooij, Kak Khee Yeung, Aart J. Nederveen, Oliver J. Gurney‐Champion

**Affiliations:** ^1^ Radiology and Nuclear Medicine Amsterdam University Medical Center Amsterdam the Netherlands; ^2^ Amsterdam Cardiovascular Sciences Amsterdam the Netherlands; ^3^ Cancer Center Amsterdam Imaging and Biomarkers Amsterdam the Netherlands; ^4^ Vascular Surgery Amsterdam University Medical Center Amsterdam the Netherlands

## Abstract

**Purpose:**

To develop and validate a protocol for simultaneous volumetric T1 and T2 mapping with blood‐ and fat‐suppression, and apply it to patients with abdominal aortic aneurysms (AAA) to assess tissue characteristics.

**Methods:**

A spoiled gradient echo (SPGR) variable flip angle (VFA) acquisition was modified with preparation modules to suppress blood and fat signals. To account for the additional T1 and T2 weighting introduced by these preparation blocks, a dedicated signal model was developed, enabling accurate simultaneous T1 and T2 quantification. The method was validated through Bloch simulations and phantom experiments. Subsequently, the protocol was applied to 20 AAA patients.

**Results:**

Bloch simulations perfectly overlaid the signal model, validating the model. In phantom experiments, the bias for T1 was reduced from −781 to 100 ms when incorporating the signal model, and the bias for T2 was −7 ms. In vivo application in 20 AAA patients demonstrated the feasibility of generating volumetric T1 and T2 maps of the aortic vessel wall with blood‐ and fat‐suppression.

**Conclusion:**

The proposed method enables simultaneous T1 and T2 mapping with blood‐ and fat‐suppression in AAA patients. Validation in simulations and phantom experiments confirmed accuracy, while in vivo experiments demonstrated technical feasibility, providing a foundation for future clinical studies on vessel wall tissue characterization.

## 1 Introduction

An abdominal aortic aneurysm (AAA) is a potentially life‐threatening condition characterized by the progressive dilation of the abdominal aorta. If left untreated, AAAs can rupture, leading to high mortality rates [1]. Although elective surgical repair can prevent rupture, it is an invasive procedure with substantial risks [2]. Therefore, accurate estimation of AAA progression would be beneficial to identify which patients are most likely to benefit from intervention and which may be safely monitored.

Currently, the primary criterion for assessing AAA rupture risk is the maximum aortic diameter and growth [3]. While diameter is an easily accessible macrostructural measure, it does not capture the underlying microstructural changes within the aneurysm wall, such as inflammatory infiltration. These microstructural alterations might be involved in wall weakening and rupture risk [4, 5, 6]. The reliance on diameter alone, therefore, potentially overlooks important biological processes that may better predict clinical outcomes.

T1 and T2 relaxometry offer the potential to non‐invasively assess the tissue composition of the aortic wall and intraluminal thrombus (ILT). While T1 and T2 relaxation are time‐constant measurements, they are influenced by relevant microstructural factors. For example, T1 is affected by fibrosis, lipid content, and extracellular volume [7], and T2 correlates with tissue water content, edema and inflammation [8]. By capturing surrogate markers for these microstructural properties, relaxometry has the potential to provide imaging biomarkers that improve risk stratification beyond anatomical measurements.

However, technical challenges limit the application of T1 and T2 relaxometry in AAAs. The high signal intensity of flowing blood (“bright blood”) inside the aorta and fatty tissue surrounding the aorta complicates accurate AAA delineation. Furthermore, the thinness of the aortic wall creates partial volume effects with these bright signals that confound precise mapping. Finally, respiratory motion during acquisition also introduces artifacts from these bright signals into the aortic wall, further impacting image quality, T1 and T2 quantification, and reproducibility. Together, these factors make reliable relaxometry assessment of AAA structures challenging.

Several solutions exist to overcome these challenges. Blood suppression (also known as black blood) can be achieved with various techniques, such as dephasing moving spins, presaturating inflowing blood, or nulling the signal with double inversion. These approaches have been shown to enhance vessel wall visibility [9, 10]. Fat suppression methods, such as spectral fat saturation [11] and Dixon techniques [12], have been successfully applied in multiple body regions to improve overall image quality [13]. Motion compensation or correction strategies, including navigator gating and motion‐resolved reconstructions, help reduce respiratory motion artifacts [14, 15, 16, 17].

However, combining these techniques is challenging because blood‐ and fat‐suppression techniques often disrupt the steady‐state magnetization required for accurate and efficient T1 mapping based on a variable flip angle (VFA) approach. Moreover, these suppression pulses introduce additional T1 or T2 weighting depending on the pulses used, which can complicate quantitative measurements. Adding motion compensation further increases sequence complexity and reconstruction demands. As a result, no protocol (based on a VFA approach) to date has integrated blood suppression, fat suppression, and motion compensation for abdominal aortic relaxometry, and no model exists to describe such VFA data and obtain accurate T1 and T2 values.

To address these limitations, we developed a relaxometry protocol that integrates blood suppression, fat suppression, and motion compensation within a 3D VFA acquisition. Recognizing the challenges posed by non steady‐state conditions and additional contrast weighting, we also implemented a dedicated signal model tailored to these conditions. The technique was validated in a phantom study and applied in vivo in 20 patients with AAAs. Derived T1 and T2 values were compared against AAA diameter.

## 2 Theory

Under conditions of perfect spoiling and steady state magnetization, the signal (*S*) from a spoiled gradient echo (SPGR) acquisition can be described by the Ernst equation:

(1) $$S=M_{0}\frac{\sin\left(\alpha \right)\left(1-E_{1}\right)}{1-\cos\left(\alpha \right)E_{1}}$$

where *M*_0_ is the equilibrium magnetization, *α* is the flip angle (FA), and $E_{1}=\exp\left(-\frac{\mathrm{TR}}{T1}\right)$ (TR is the repetition time, and T1 is the longitudinal relaxation time). In VFA T1 mapping, multiple images at different FAs are acquired, and T1 is estimated by fitting Equation (1).

**FIGURE 1:**
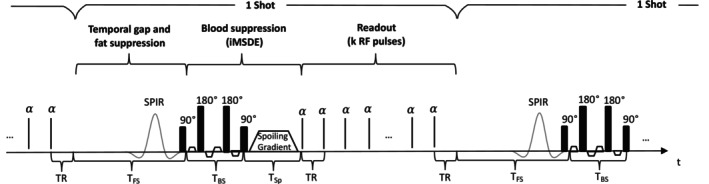
The I‐SPGR sequence used: Each imaging block (shot) starts with a preparation block in which blood and fat suppression is applied (SPIR and iMSDE), followed by a readout block in which *k* gradient echoes are acquired.

To enable blood‐ and fat‐suppression, the SPGR sequence needs to be interleaved with preparation pulses. In the resulting interleaved spoiled gradient echo (I‐SPGR) sequence, the signal evolution differs from that predicted by the standard SPGR model due to the introduction of preparation blocks followed by readout blocks (Figure 1). Our I‐SPGR sequence consists of repeated imaging blocks (hereafter defined as shots) with each consisting of:

- *Preparation blocks*
  ∘ *Temporal gap and fat suppression*: To comply with specific absorption rate (SAR) limitations, a temporal gap is introduced before the fat suppression module. During this gap, no RF pulses are applied, and only longitudinal (T1) relaxation occurs. Hence, this pause introduces additional T1 weighting. Following the temporal gap, fat suppression is performed using a spectral presaturation with inversion recovery (SPIR) pulse, which selectively excites off‐resonance fat spins and spoils their remaining transverse magnetization. By exciting the spins with the correct FA, fat signal can be suppressed at a carefully timed point during readout, during which the center of k‐space is typically measured [18]. The on‐resonance signals of interest are unaffected by the SPIR pulse and only experience T1 relaxation during this time. Therefore, the total duration of the combined temporal gap and SPIR pulse is denoted as *T*_*FS*_.
  ∘ *Blood suppression*: Blood suppression is achieved using an improved motion‐sensitized driven equilibrium (iMSDE) preparation block [19]. This block temporarily flips the entire magnetization into the transverse plane (duration in transverse plane given by *T*_*BS*_) and uses a balanced gradient scheme to achieve signal suppression of flowing spins in larger vessels. This is followed by a period of spoiling (*T*_*Sp*_). When the magnetization is in the transverse plane, it is affected by T2 relaxation, whereas during the spoiling period, it is affected by T1 relaxation. The iMSDE pulse substantially reduces signal from moving spins while allowing static spins to evolve according to their T1 and T2 properties.
- *Readout block*: A series of *k* RF pulses and k‐space readouts are played out (including gradient and RF spoiling).

The preparation pulses introduce a transient signal evolution during the readout phase, making the SPGR steady‐state equation obsolete. Starting from the work of Qi et al. [20] and our previous work [21], we derived a signal model that accounts for both T1 and T2 effects of the preparation blocks to enable accurate simultaneous T1 and T2 mapping. After a sufficient number of shots, the signal is assumed to reach a semi steady state, in which the signal evolves similarly for each readout block, but changes throughout the readout block. With transverse signal spoiling, we only need to keep track of the longitudinal signal. During each readout, the longitudinal signal is only affected by the RF pulse and longitudinal T1 relaxation. The longitudinal magnetization at the *n*th + 1 readout can then be expressed with the following recursive equation:

(2) $$\begin{array}{cc} M_{n+1} & =M_{n}\cos\left(\alpha \right)+\left(M_{0}-M_{n}\cos\left(\alpha \right)\right)\left(1-E_{1}\right)\\ & =M_{n}E_{1}\cos\left(\alpha \right)+M_{0}\left(1-E_{1}\right) \end{array}$$

This can be rewritten into:

(3) $$M_{n}=M_{1}{\left(E_{1}\cos\left(\alpha \right)\right)}^{n-1}+M_{0}\left(1-E_{1}\right)\sum_{a=0}^{n-2}E_{1}^{a}\cos^{a}\left(\alpha \right)$$

For all *n* ϵ [2, *k*]. *M*_*n*=1_ is the longitudinal magnetization just before the start of the readout block, which is identical to the longitudinal magnetization at the end of the preparation block: *M*_*End‐prep*_ = *M*_*n*=1_. Using the geometric series $\left(\sum_{a=0}^{b}r^{a}=\frac{1-r^{b+1}}{1-r}\right)$, *M*_*n*_ can then be expressed by:

(4) $$M_{n}=M_{\mathrm{End}-\text{prep}}{\left(E_{1}\cos\left(\alpha \right)\right)}^{n-1}+M_{0}\left(1-E_{1}\right)\frac{1-{\left(E_{1}\cos\left(\alpha \right)\right)}^{n-1}}{1-E_{1}\cos\left(\alpha \right)}$$

The preparation block starts after the last readout. With *k* pulses in the readout block (*n* ϵ [1, *k*]), the longitudinal magnetization just before the preparation block is *M*_*k*+1_. The first part of the preparation block is modeled by additional T1 relaxation (temporal gap due to SAR cooldown and off‐resonance fat suppression). The longitudinal magnetization just before the beginning of the blood suppression block can then be expressed by:

(5) $$M_{\text{Blood}-\text{suppression}}=M_{k+1}+\left(M_{0}-M_{k+1}\right)\left(1-E_{\mathrm{FS}}\right)$$

With $E_{\mathrm{FS}}=\exp\left(-\frac{T_{\mathrm{FS}}}{T1}\right)$. During the blood suppression pulse, the longitudinal magnetization is flipped into the transversal plane, refocused two times and then flipped back into longitudinal direction after *T*_*BS*_. During the time in the transversal plane, T2 relaxation occurs, so the longitudinal relaxation just before the spoiling gradients is given by:

(6) $$M_{\text{Spoil}}=M_{\text{Blood}-\text{suppression}}\;E_{\mathrm{BS}}$$

with $E_{\mathrm{BS}}=\exp\left(-\frac{T_{\mathrm{BS}}}{T_{2}}\right)$. Defining $E_{\mathrm{Sp}}=\exp\left(-\frac{T_{\mathrm{Sp}}}{T1}\right)$ and combining Equations ((4), (5), (6)) we can derive the longitudinal magnetization before the first RF pulse (*M*_*End‐prep*_ = *M*_*n*=1_):

(7) $$\begin{array}{cc} M_{n=1} & =M_{\mathrm{End}-\text{prep}}=M_{\text{Spoil}}+\left(M_{0}-M_{\text{Spoil}})(1-E_{\mathrm{Sp}}\right)\\ & =M_{0}\frac{\left(1-E_{\mathrm{Sp}}\right)+\left(1-E_{\mathrm{FS}}\right)E_{\mathrm{Sp}}E_{\mathrm{BS}}+E_{\mathrm{Sp}}E_{\mathrm{BS}}E_{\mathrm{FS}}\left(1-E_{1}\right)\frac{1-{\left(E_{1}\cos\left(\alpha \right)\right)}^{k}}{1-E_{1}\cos\left(\alpha \right)}}{\left[1-E_{\mathrm{Sp}}E_{\mathrm{BS}}E_{\mathrm{FS}}{\left(E_{1}\cos\left(\alpha \right)\right)}^{k}\right]} \end{array}$$

Equation (7) can then be inserted into Equation (4), which gives an equation describing the longitudinal magnetization evolution right before each RF pulse during the shot. Hence, for a block with k RF pulses, the signal after RF pulse *n* is given by:

(8) $$\begin{array}{cc} S_{n} & =\sin\left(\alpha \right)M_{0}[\left(1-E_{1}\right)\frac{1-{\left(E_{1}\cos\left(\alpha \right)\right)}^{n-1}}{1-E_{1}\cos\left(\alpha \right)}\\ & \;+\frac{\left(1-E_{\mathrm{Sp}}\right)+\left(1-E_{\mathrm{FS}}\right)E_{\mathrm{Sp}}E_{\mathrm{BS}}+E_{\mathrm{Sp}}E_{\mathrm{BS}}E_{\mathrm{FS}}\left(1-E_{1}\right)\frac{1-{\left(E_{1}\cos\left(\alpha \right)\right)}^{k}}{1-E_{1}\cos\left(\alpha \right)}}{\left[1-E_{\mathrm{Sp}}E_{\mathrm{BS}}E_{\mathrm{FS}}{\left(E_{1}\cos\left(\alpha \right)\right)}^{k}\right]}\\ & \;{\left(E_{1}\cos\left(\alpha \right)\right)}^{n-1}] \end{array}$$

Equation (8) describes the measured signal in a blood‐ and fat‐suppressed I‐SPGR sequence for each of the readout segments as a function of the tissue's T1 and T2 values, and several acquisition settings: FA *α*, fat suppression duration $T_{\mathrm{FS}}$, blood suppression duration $T_{\mathrm{BS}}$, spoiling duration $T_{\mathrm{Sp}}$, repetition time (*TR*) and shot length *k*.

Before image acquisition, parameters such as FA and TR are predefined, and the resulting image intensities are governed by the tissue‐specific properties T1, T2, and M0. By acquiring multiple images with systematically varied acquisition settings, such as FA and blood suppression preparation time, the signal response becomes sensitive to tissue T1 and T2 properties. In this work, we vary the FA and the duration of the blood suppression preparation pulse, as these parameters primarily influence T1 and T2 weighting, respectively. The resulting series of images, in combination with the derived signal model, is then used in a joint fitting process to estimate T1, T2, and M0 simultaneously.

## 3 Methods

### 3.1 Simulations: Validation of Signal Equation

To validate Equation (8), we implemented and performed Bloch simulations based on the framework described by Preibisch and Deichmann [22]. First, for each readout, an instantaneous RF excitation with FA *α* was applied to the magnetization vector. The resulting transverse magnetization immediately after the RF excitation was stored and compared to the predictions of the analytical signal equation. Then longitudinal relaxation during the TR was modeled using T1 decay, and the transverse magnetization was set to zero, assuming perfect spoiling. After k RF pulses, the first temporal gap ($T_{\mathrm{FS}}$) was simulated using T1 and T2 relaxation only. Next, the iMSDE blood suppression preparation was modeled by applying T2 relaxation for the duration of $T_{\mathrm{BS}}$ to the longitudinal magnetization. This was followed by T1 relaxation during $T_{\mathrm{Sp}}$ before simulating the next readout block. The simulation was run for isochromats with T1 values of 500 and 1500 ms, in combination with T2 values of 25 and 50 ms. Acquisition settings were: TR = 5 ms, FA = 15°, *k* = 20, *T*_*FS*_ = 50 ms, *T*_*BS*_ = 12 ms, and *T*_*Sp*_ = 6 ms.

To investigate the effects of imperfect spoiling using RF and gradient spoiling, we performed additional simulations, detailed in Supporting Information S1.

We also conducted a sensitivity analysis to examine the impact of noise in the acquired data on the estimated T1, T2, and M0 values. A full description of the methods and results is provided in Supporting Information S2.

### 3.2 Data Acquisition

#### 3.2.1 Phantom Data

To validate the proposed technique, a Gold Standard Phantom MultiSample 120S phantom (Gold Standard Phantoms, Sheffield, United Kingdom) was scanned. It contains eight vials filled with different MnCl_2_ solutions (without fat) and T1 and T2 reference values provided in the manufacturer's manual. T1 values range from 236 to 2013 ms and T2 values range from 15 to 328 ms. Data were acquired on a 3 T Philips Ingenia Elition X system (Philips, Best, The Netherlands; Software version 11.1) using a 16‐channel head coil. Phantom data were collected using an SPGR sequence with six different FAs (25°, 20°, 15°, 10°, 5°, 2°, similar to [21]), and an I‐SPGR sequence with blood‐ and fat‐suppression using four FAs (25°, 20°, 10°, 4°). FA 15° was skipped to avoid redundancy with a dynamic contrast enhanced (DCE) scan performed with FA 15° in the same study; FA 2° provided insufficient signal due to the additional T2 weighting and was combined with FA 5°, resulting in a FA of 4°. *T*_*BS*_ of 13 ms was the shortest available on the scanner, and *T*_*BS*_ longer than 35 ms yielded too little signal; 20 ms was therefore selected as an intermediate *T*_*BS*_. As an FA of 10° was closest to the Ernst angle for most T1s, this FA was used when varying *T*_*BS*_. Acquisition parameters are summarized in Table 1. Phantom data were acquired on two consecutive days, and measurements were repeated on the first day to evaluate both reproducibility and repeatability.

**TABLE 1:** Acquisition settings used for phantom and in vivo scans.

	Phantom		In vivo
Seq. title	SPGR VFA	I‐SPGR VFA	I‐SPGR VFA
TR	6.6 ms		3.5 ms
TE	1.2 ms		
FA	25°, 20°, 15°, 10°, 5°, 2°	25°, 20°, 10°, 4°	
FOV (RL, AP, FH)	420 × 268 × 100 mm^3^		420 × 268 × 100 mm^3^ or 500 × 329 × 100 mm^3^
Acquired voxelsize	1.5 × 1.5 × 4 mm^3^		1.5 × 1.5 × 4 mm^3^ or 1.8 × 1.8 × 4 mm^3^
Reconstructed voxelsize	0.98 × 0.98 × 4 mm^3^		0.98 × 0.98 × 4 mm^3^ or 1.16 × 1.16 × 4 mm^3^
RF pulses per shot	NA	30	
Duration blood suppression	NA	13 ms for each FA, for FA 10° additionally 20 and 35 ms	
Duration fat suppression	NA	110 ms^a^	202 ms
Duration spoil	NA	10 ms for $T_{\mathrm{BS}}$ = 13 ms; 14 and 21 ms for $T_{\mathrm{BS}}$ = 20 and 35 ms	
Acquisition time	5 min	8 min	

^a^ For phantom experiment, the fat suppression duration was chosen such that the total shot length was the same as in vivo, compensating for the longer TRs.

#### 3.2.2 In Vivo Data

Cross sectional in vivo I‐SPGR data were acquired from 20 AAA patients as part of a prospective single‐center study (ClinicalTrials.gov ID: NCT05976711) which received approval from the Amsterdam UMC medical ethics board in accordance with the Dutch Medical Research Involving Human Subjects Act (WMO). In addition to the patient cohort, same‐day in vivo repeatability experiments were performed in a separate group of seven healthy volunteers. All participants provided written informed consent and patients were recruited with an asymptomatic AAA of at least 30 mm in diameter, as clinically determined based on CT, US, or MRI. Patient exclusion criteria were severely impaired renal function, supra‐ or pararenal AAA, previous AAA repair, untreated cardiac arrhythmias, inflammatory, infectious, or mycotic AAA, untreated vasculitis, or a connective tissue disorder. Scanning was performed using a 32‐channel body coil on the same scanning system as mentioned above (software version 5.9). Acquisition settings (such as TR, duration of preparations, k‐space sampling scheme) were chosen heuristically with a focus on achieving the best possible image quality while staying within SAR boundaries and an acquisition time of approximately 1 min for each FA and *T*_*BS*_ pair. Different combinations of TR, number of RF pulses per shot, *T*_*FS*_, *T*_*BS*_, and k‐space sampling patterns were tested during protocol development. Final settings in Table 1 were chosen heuristically based on simulated signal curve shapes, VFA literature and visual assessment of aneurysm visibility, fat suppression around the aneurysm, effective blood suppression, and overall image quality. The FOV was adjusted according to the patient's size in the AP direction, using either a smaller or larger FOV as needed. Acquired and reconstructed voxel sizes were chosen accordingly, while maintaining the same acquisition matrix. B0 and B1 shim boxes were placed on the aneurysm.

For all experiments, B1^+^ maps were acquired in a free breathing scan using the Actual Flip Angle Imaging method (AFI) [23] with a resolution of 6 × 6 × 12 mm^3^, a FA of 60°, and a dual‐TR scheme (TR = 30 ms, TR extension = 120 ms). A gradient spoiling factor of 40 (=40 times Philips default) was used for all phantom scans and a factor of 10 for in vivo acquisitions. In phantoms, a spoiling factor of 10 proved insufficient, whereas a factor of 40 produced reliable B1^+^ maps. In vivo, however, spoiling factors of 10 or 40 yielded comparable results. As the patient study was started with a factor of 10, this setting was maintained throughout. The spoiling gradients were applied in all three spatial directions to ensure effective dephasing of residual transverse magnetization and to suppress echoes that may form between interleaved TR periods. Details on how B1^+^ maps were used to correct for spatial transmit field variations are provided in Section 3.3.

#### 3.2.3 K‐Space Sampling

As shown in Equation (8), the signal evolution changes during each readout shot but repeats with every shot. This disruption is strongest in the first readouts and diminishes later in the shot, as the signal approaches a steady state. At the same time, the effect of preparation pulses (blood and fat suppression in this case) is strongest at the start of the shot. The central region of k‐space reflects the point at which most of the image contrast is determined. Therefore, to make optimal use of the preparation, the signal immediately after preparation is used to sample central k‐space, while signals from later readouts—when the magnetization is closer to steady state—are used to sample peripheral k‐space.

**FIGURE 2:**
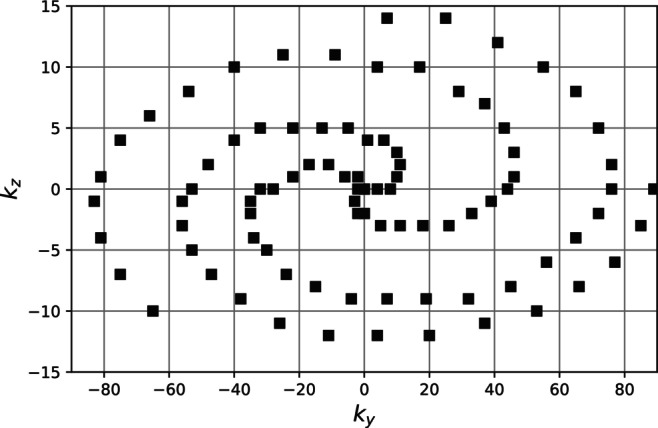
K‐space sampling pattern showing three example pseudo‐spiral trajectories. Each spiral consists of 30 readouts and corresponds to one readout block. The spirals are rotated by the golden angle after each block to ensure dense sampling of the central k‐space.

To ensure effective k‐space coverage, a pseudo‐spiral‐like trajectory was employed [24, 25, 26]: k‐space is traversed in a spiral‐like manner on a Cartesian grid, with *k*_*x*_ as the frequency encoding direction, and *k*_*y*_ and *k*_*z*_ as the phase and slice encoding directions, respectively. The spiral is defined parametrically as $r=-x^{2}+2x$ with x∈[0,1] and angular coordinate φ ranging from 0 to 2*π*. This spiral is then mapped onto the Cartesian (*k*_*y*_, *k*_*z*_) plane (Figure 2), using a matrix size of 178 × 30. Each spiral arm corresponds to one shot and includes 30 sampled points. After each shot, the spiral is rotated by a golden angle of 137° [27], which ensures dense coverage of central k‐space while allowing peripheral regions to remain undersampled.

This pseudo‐spiral sampling pattern retains some of the advantages of spiral acquisitions—such as robustness to motion and incoherent undersampling artifacts beneficial for compressed sensing (CS)—while maintaining Cartesian sampling compatibility. It also supports respiratory‐compensated reconstruction (see Section 3.3) and facilitates scan acceleration.

### 3.3 Data Processing

After data acquisition, raw data were exported and reconstructed offline in MATLAB 2021b (MathWorks, Natick, MA) in combination with ReconFrame 5.4.1 (Gyrotools, Zurich, Switzerland). Image reconstruction employed a nonlinear compressed sensing algorithm [28] implemented in the Berkeley Advanced Reconstruction Toolbox (BART) [29].

For the in vivo data, three respiratory‐resolved images were reconstructed based on the respiratory signal extracted from a respiratory belt. The image corresponding to the expiratory phase was used for further processing, while the other two respiratory states were discarded.

Voxel‐wise actual FA *α*_*actual*_ were derived from B1^+^ maps acquired during the imaging protocol: 

(9)

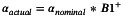


where *α*_*nominal*_ was the set FA. Before fitting, the B1^+^ maps were smoothed using a three‐dimensional Gaussian filter to reduce local noise and improve the robustness of the correction.

For the phantom data, the vials in the central slice were manually segmented using MATLAB. For the patient data, segmentations including the aortic wall and ILT (if present) were generated using nnInteractive [30] with napari [31] based on the image with the highest contrast, usually the image with a FA of 10°. Segmentations were performed by WS, researcher with 4 years of experience in AAA qMRI imaging. For the healthy volunteers, the healthy aorta was too small to reliably segment and hence the psoas were segmented to assess repeatability, instead. In all segmented regions of interest (ROI), T1 and T2 values were then estimated on a voxel‐by‐voxel basis by fitting the I‐SPGR equation (Equation 9) with SciPy's curve_fit function [32]. Fitting boundaries were set to T1 ϵ [100, 3000] ms and T2 ϵ [20, 300] ms. As image contrast is predominantly defined in the center of k‐space, which was obtained first in each shot, we used a fixed value of *n* = 1 in the model to fit the data.

For the phantom data, mean T1 and T2 values were estimated for each vial and scan. These T1 and T2 values were compared against the manual reference values. Additionally, the I‐SPGR dataset was fitted using the standard SPGR equation (Equation 1) to assess potential bias introduced when neglecting the effects of the preparation pulses.

For the in vivo data, T1 and T2 values were fitted within the segmented regions using the methods described above, resulting in volumetric T1 and T2 maps for each patient. Aneurysm diameter was measured based on Dixon images acquired during the same imaging session, using a commercial implementation of the 2‐point Dixon method [33] (diameter measured by WS). ILT, if present, was considered as being part of the lumen.

### 3.4 Statistics and Analysis

Bloch simulations were assessed visually. We first verified that the simulations match the analytical solution. Then, we characterized the signal behavior throughout the shots. For the phantom experiments, the estimated T1s and T2s were plotted as a function of the ground‐truth reference values.

Phantom repeatability was evaluated using the repeated scans acquired at 1 day whereas reproducibility compared the scans from separate days. Measurement differences were visualized using Bland–Altman plots and quantified by the within‐subject coefficient of variation (wCV).

For healthy volunteer data, also Bland–Altman plots were generated and wCVs were calculated.

In patients, to investigate the relationship between quantitative relaxation values and maximum aneurysm diameter, median T1 and T2 values were extracted and correlated against the current clinical gold standard for assessing aneurysm progression: the maximum aneurysm diameter.

Depending on the distribution of the fitted median T1/T2 values and aneurysm diameter (assessed using the Shapiro–Wilk test, with *p* < 0.05 indicating deviation from normality), either Pearson's correlation coefficient or Spearman's rank correlation coefficient was used. A *p* < 0.05 was considered statistically significant for all tests.

## 4 Results

### 4.1 Simulations: Validation of Signal Equation

**FIGURE 3:**
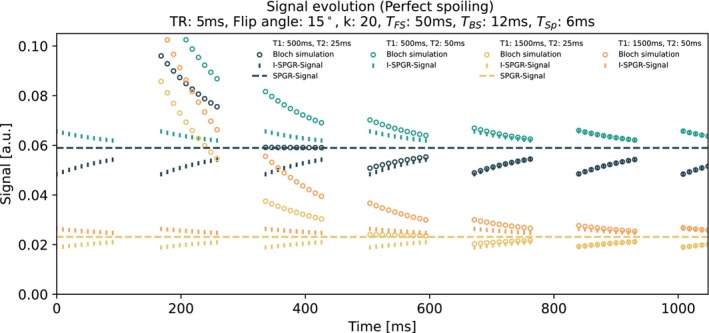
Validation of the signal model by comparing Bloch simulations to the derived steady‐state equation (Equation 8). Shown are the simulated signals from a Bloch simulation (circles), the derived analytical signal model (vertical lines), and, as a reference, the steady‐state signal from a conventional SPGR sequence (Equation 1, dashed horizontal line). Simulations used four representative T1/T2 pairs, with T1 values of 500 and 1500 ms and T2 values of 25 and 50 ms. For visualization purposes, only every second simulated signal point is plotted. After 1 s, all signals have converged to steady state, and the Bloch simulations overlay with our steady‐state equation.

Initially, the signals calculated by the perfect spoiled Bloch simulation deviate from the derived analytical model, as the magnetization has not yet reached the transient steady state (Figure 3). However, after roughly 1 s, all Bloch‐simulated signals overlap with the I‐SPGR signal model, validating the derived model under perfect spoiling conditions. The exact time to reach this steady state depended on the T1 and T2 simulated.

The signal evolution within each shot approaches the steady‐state SPGR signal. After preparation pulses are applied, this convergence resets, causing a deviation that gradually diminishes across the readouts within a shot. Additionally, depending on the T1 and T2 values, the I‐SPGR and Bloch‐simulated signals approach the SPGR steady‐state either from a higher or lower signal amplitude, reflecting different relaxation dynamics during the readout phase.

#### 4.1.1 Phantom Data

**FIGURE 4:**
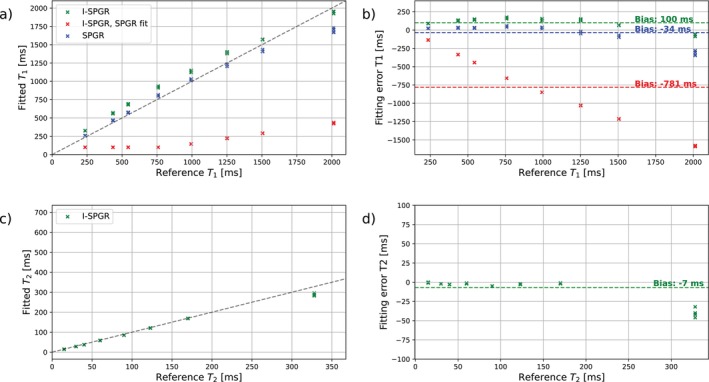
Fitted T1 and T2 values compared to reference values using SPGR and I‐SPGR measurements. (a) Fitted T1 values versus reference T1 for SPGR (blue) and I‐SPGR (green), and I‐SPGR data fit with the SPGR model (red). (b) T1 fitting error. (c) Fitted T2 values from I‐SPGR data versus reference T2 values. (d) T2 fitting error.

Naively using the SPGR equation on blood‐ and fat‐suppressed I‐SPGR acquisition results in a very large bias for T1 estimates of −781 ms (red line Figure 4a,b). Using our suggested equation substantially reduces these biases to +100 ms (green line) and brings them close to regular SPGR acquired data (−34 ms, blue line). For T2 mapping, based on the I‐SPGR data, the fitted values closely align with the reference measurements (deviations from 0 ms up to −48 ms), demonstrating a minimal mean error of −7 ms (green line Figure 4c,d).

These findings show feasibility of acquiring blood‐ and fat‐suppressed pseudo‐spiral MRI and validate the use of our novel I‐SPGR equation for T1 and T2 parameter mapping.

Across all sequences, T1 and T2 measurements showed high same‐day repeatability and between‐day reproducibility, with small mean differences and wCV values ≤ 2% (details in the Supporting Information S2).

#### 4.1.2 In Vivo

**FIGURE 5:**
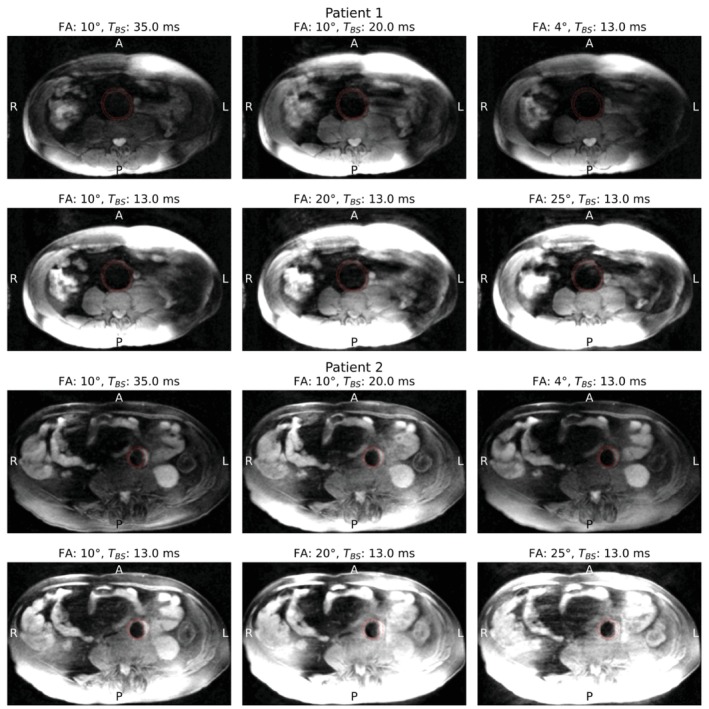
Central slices of reconstructed images for two representative patients. The red lines indicate the segmented aneurysm boundaries (inner and outer). Due to the blood suppression, the lumen does not have any signal. A single intensity scale was applied across all flip angles, resulting in saturation of high‐signal regions in some images, particularly in posterior regions.

Reconstructed central‐slice images for two representative patients acquired using six different combinations of flip angles and blood suppression preparation times are shown in Figure 5. Red contours indicate the segmented inner and outer aneurysm boundaries. Due to the applied blood suppression, the lumen has almost no signal. Also, all fat close to the vessel is suppressed by the used fat suppression pulse. These two pulses made it possible to distinguish between the lumen and aortic wall. For the first patient, it is clear that there was sometimes a large signal at the anterior left edge of the patient, presumably due to fat suppression failure in that region (B0 shim box focused on the AAA), as well as coils being more sensitive there. The aneurysm of patient two is smaller in comparison to patient one's aneurysm. Residual streaking artifacts were likely related to a combination of k‐space undersampling and remaining respiratory motion within the expiration‐only state. In some cases, artifacts were predominantly observed in the anterior region and did not directly overlap with the aortic wall (patient one, Fa 20° and 25°), whereas for patient two FA 25° mild streaking artifacts overlapped the aneurysm.

**FIGURE 6:**
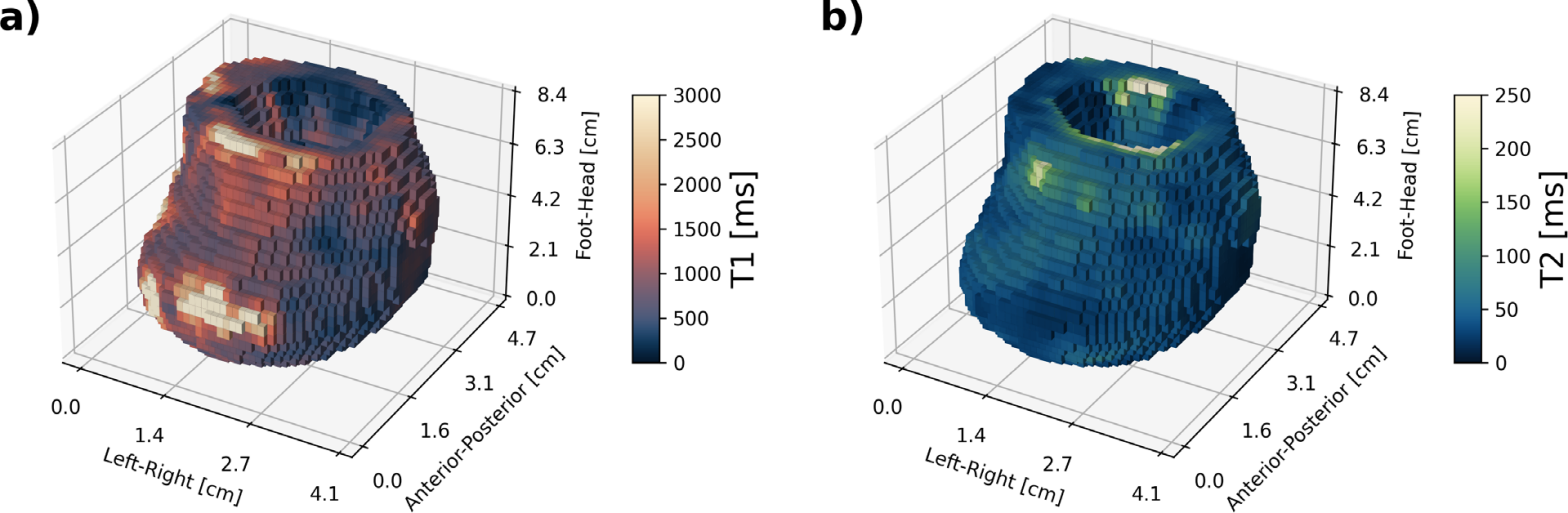
Volumetric T1 and T2 parametric maps of an individual patient, showing smooth and continuous spatial variation across the segmented aortic volume. T1 median is 891 ms and median T2 is 46 ms. High T1 values appear predominantly on the anteroinferior side of the vessel wall, while highest T2 values appear at the posterosuperior side.

Volumetric T1 and T2 maps of one representative patient are shown in Figure 6. The large‐scale variations between vessel sub‐regions are larger than the voxel‐to‐voxel variation (noise‐induced variation). For this patient, the median fitted T1 value was 891 ms (interquartile range (IQR): 723–1112 ms) and the fitted median T2 value 46 ms (IQR: 36–60 ms). Fitted values vary across different regions; for example, for this patient, the highest T1 values were observed in the anteroinferior region of the vessel wall (T1 values reached the upper fitting boundaries here), while the lowest appeared on the anterior (right) and posterior sides. The highest fitted T2 values were found in the posterosuperior region (also reaching the maximum fitting limit), while the lowest T2 values were observed on the (inferior) right side. The occurrence of a large range of fitted values throughout the vessel walls, as well as the overall smooth appearance of the maps, was consistent across patients. However, the specific regions where these extreme values occurred varied between patients.

**FIGURE 7:**
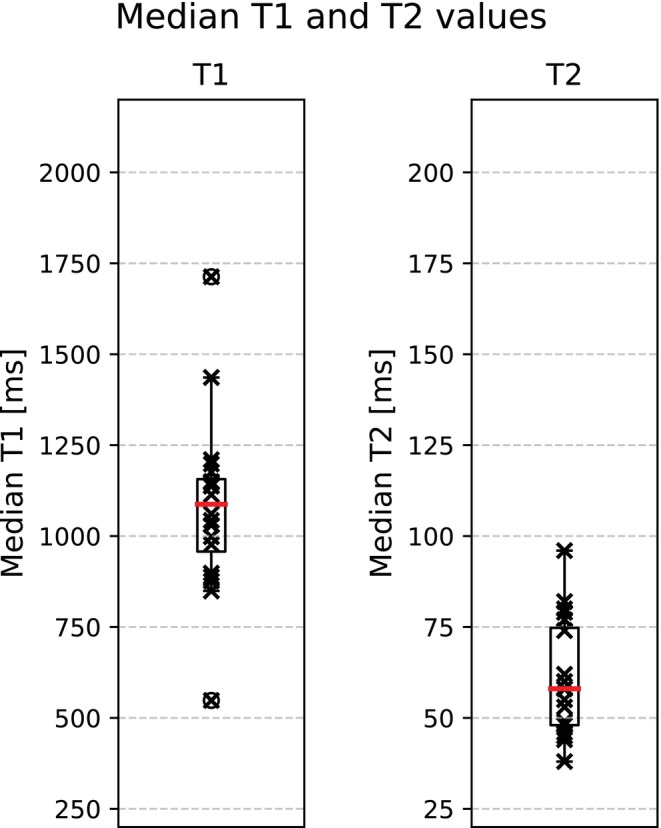
Comparison of median T1 and T2 values. Left: Boxplot of median T1 values across patients. The red horizontal line indicates the group median (1087 ms), and the black box represents the IQR (957–1156 ms for T1). Individual patient values are shown as black crosses. Right: Boxplot of median T2 values across patients, with the group median marked in red (58 ms, IQR: 48–74 ms).

**FIGURE 8:**
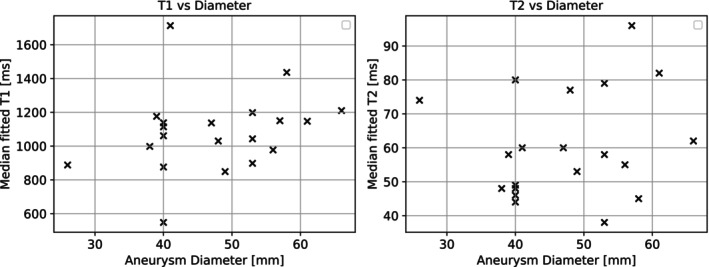
Scatter plots showing the relationship between maximum aneurysm diameter and median fitted relaxation times: T1 (left) and T2 (right). Aneurysm diameters ranged from 26 to 66 mm. No correlation was observed between diameter and T1 (Pearson's *ρ* = 0.24) or T2 (Spearman's *ρ* = 0.18) values.

The distribution of median T1 and T2 relaxation values across patients is illustrated in Figure 7. Aneurysm diameter, T1 and T2 were normally distributed. No significant correlation was found between aneurysm diameter and median relaxation times (Figure 8; T1: *ρ* = 0.24, *p* = 0.29; T2: *ρ* = 0.18, *p* = 0.44).

In healthy volunteers, a stable same day in vivo repeatability of T1 and T2 measurements using the I‐SPGR sequence was found for the psoas. Within‐subject coefficient of variation was 0.11 and 0.05 for T1 and T2, with T1 values ranging from 1387 to 1756 ms and mean T2 values ranging from 32 to 51 ms (details in Supporting Information S3).

## 5 Discussion

We present a novel blood‐ and fat‐suppressed, VFA‐based, pseudo‐spiral readout and motion‐compensated I‐SPGR approach for simultaneous T1 and T2 mapping in the vessel wall and applied it to AAA patients. Imaging the vessel wall of AAAs and performing T1 and T2 mapping presented several challenges, which we overcame with our protocol: First, the used suppression pulses enabled delineation of the aortic wall from surrounding tissues and the lumen, independent of ILT presence. Our free breathing sequence is also faster than previous protocols [34, 35]. Additionally, to address the non steady‐state behavior of the I‐SPGR sequence and to avoid systematic errors, we developed a dedicated signal model that enables unbiased simultaneous T1 and T2 fitting (when compared to the standard VFA signal model). Simulations and phantom experiments confirm the accuracy and precision of our method. With our method, we quantified T1 and T2 in the aortic walls of 20 AAA patients. In the future, these parameters may give insight into typical microstructural properties of the aneurysm wall and help identify patients at risk of rupture.

Our study is the first to enable simultaneous T1 and T2 mapping in the abdominal aortic wall, overcoming the specific challenges of this anatomy. Studies have applied relaxometry in vessel walls in general, but especially in the abdominal aortic wall, the literature is limited. Early work demonstrated the feasibility of noninvasive MR evaluation of aortic wall pathology using conventional non‐quantitative MRI in the thoracic aorta [36]. Building on this foundation, more recent studies have advanced toward quantitative mapping in the carotid arteries. Coolen et al. [34] and Xu et al. [35] performed three‐dimensional simultaneous T1 and T2 (and T2*) mapping in the carotid artery using comparable sequences (I‐SPGR with iMSDE and fat suppression), but with key differences: they employed dummy pulses during each shot to ensure steady‐state signal at the end of each shot resulting in longer acquisition times (∼170 and ∼140 s per acquired image compared to 85 s in our study). Moreover, carotid arteries are less affected by respiratory motion. Our approach models the signal throughout the entire shot, eliminating the need for dummy pulses and enabling faster scans. Additionally, we correct for respiratory motion and use a shorter TR (3.5 ms compared to 10 and 8 ms). Our approach overcomes previous technical limitations, especially those for abdominal imaging, and paves the way for personalized assessment of vessel wall health in AAA patients.

The design of our acquisition was guided by the need for a sequence suitable for both quantitative T1/T2 mapping and a subsequent 3D DCE scan of the entire aneurysm. We therefore adopted a VFA‐based approach, which provides fast 3D coverage and offers flexible timing compatible with dynamic imaging. Inversion‐recovery based T1 mapping techniques (such as [37]), were less suitable because they require sampling multiple inversion times and extend over several recovery periods, complicating their integration into a time‐efficient 3D abdominal protocol. For blood suppression, we selected iMSDE rather than flow‐independent dark‐blood preparations based on T2‐prep or magnetization transfer (such as [38]), as the latter rely on stable blood T1/T2 values, which change rapidly during DCE and would compromise suppression performance. Finally, although Dixon fat suppression is widely used, its requirement for additional echoes imposed TR constraints and, in our experience, did not consistently achieve robust fat–water separation in the abdominal aorta; SPIR yielded more reliable fat suppression in this region.

The developed sequence can be applied to DCE studies by using a single flip angle acquisition with blood and fat suppression and employing a temporally resolved reconstruction to capture dynamic contrast agent uptake. This approach offers several advantages over previous work. Nguyen et al. and Zhou et al. [39, 40, 41] conducted DCE‐MRI in AAA patients but relied on assumed pre‐contrast T1 and T2 values and excluded patients without intraluminal thrombus (ILT) due to missing blood suppression. In contrast, our protocol's blood‐ and fat‐suppression allows inclusion of all patients and enables individualized T1 and T2 mapping (before contrast administration), which is critical for accurate pharmacokinetic modeling.

Our Bloch simulations and phantom study highlight that blood‐ and fat‐suppressed VFA result in accurate T1 and T2 mapping, with an average bias of 100 ms in T1 and −7 ms in T2. Such an order of bias is typical for VFA SPGR MRI, as literature reports biases in phantoms in similar ranges (5%–32% in [42] and −15% to −7% in [43]). To our knowledge, this is the first study to perform quantitative T1 and T2 mapping in the vessel wall of AAA patients. Our median T1 value (1087 ms; 957–1156 ms IQR) and T2 (58 ms; 48–74 ms IQR) were comparable to in literature on non AAA vessel wall imaging. For example, Coolen et al. [34] and Xu et al. [35] reported simultaneous T1 and T2 mapping in the carotid vessel wall, with T1 values of 844 ms in healthy volunteers and 1041 ms in patients with atherosclerotic plaque, and T2 values of 39 and 40 ms, respectively.

Spoiling imperfections are a known challenge to VFA. Potentially, the gaps in the I‐SPGR, followed by central k‐line reading at *n* = 1 can result in less spoiling issues as some of the spoiling history will dissipate in the gaps. To explore this, Supplementary Bloch simulations that incorporated both RF and gradient spoiling were performed (Supporting Information S1). They suggest that I‐SPGR may be less sensitive to incorrect spoiling under certain settings, potentially providing a robustness advantage.

The use of the iMSDE preparation pulse, while effective for vessel wall imaging, comes at the cost of signal reduction. First, the iMSDE pulse decreases the overall signal by introducing T2 weighting. It further reduces the signal and causes signal instabilities whenever motion occurs during the iMSDE pulse. These can include cardiac and respiratory motion in all surrounding tissue. These additional T2 and motion‐related signal decays further reduce the signal‐to‐noise ratio (SNR) which negatively impacts the image quality and the precision of parameter estimation. While acknowledging the theoretical decrease in accuracy and precision, and the added decrease in SNR due to the practical implications of the iMSDE pulse when compared to conventional VFA, the latter is not an option in the abdominal aorta due to bright fat and blood obscuring the aortic wall.

To investigate how this reduction in signal affects the precision in parameter estimation, we performed additional sensitivity analyses (see Supporting Information S2). These simulations showed that the precision of T1 estimates vary with the T2 value of tissue, with poorer estimates occurring for tissue with short T2. Such short T2 values can cause a large drop in signal throughout the blood suppression pulse. As expected, they also revealed that T1 estimated from our I‐SPGR method are less precise than from conventional (SPGR) VFA. Hence, there is a reduction in precision as a result of the blood suppression pulse and fat suppression pulse that are essential for depicting the vessel wall in AAA patients. Our protocol was heuristically optimized prior to the patient study, based on visual image quality (fat and blood suppression), SAR constraints, and empirical experience. In retrospect, these may not have been the best possible acquisition settings. We hence have several suggestions that can improve the precision. Simulations suggest that increasing TR and reducing the duration of the fat suppression gap (*T*_*FS*_) could further improve precision. Additionally, modifying the k‐space filling in combination with SPIR timing and excitation angle can further enhance fat suppression efficiency. Future work could explore dictionary matching or Bloch simulations to address this.

Our study has limitations that should be considered when interpreting the results. Residual respiratory motion may have affected both the acquired images and B1^+^ maps, potentially propagating to errors in T1 and T2 quantification. The sensitivity of the iMSDE preparation pulse to B1^+^ inhomogeneity was not explicitly assessed and may influence its efficiency, despite the good observed image quality… The thin aortic wall increases susceptibility to partial volume effects from blood and surrounding tissue, potentially biasing relaxation time estimates. Finally, we lacked a gold standard reference in the AAA patients and compared T1 and T2 only with vessel diameter.

This study developed T1 and T2 as a potential marker to capture rupture risk in AAA. Currently, vessel wall diameter is the gold standard for this. If T1 and T2 purely predict rupture risk, a certain degree of correlation would be expected, however, no significant correlations were found. Possibly, T1 and T2 provide complementary microstructural information, although this cannot be shown with our current data. To investigate this, longitudinal follow‐up to study aneurysm growth and its associations with T1, T2, and diameter are needed.

## 6 Conclusion

We introduced a novel blood‐ and fat‐suppressed, motion‐compensated I‐SPGR approach for simultaneous T1 and T2 mapping of the aortic vessel wall in AAA patients, supported by a dedicated signal model tailored to account for non‐steady‐state behavior. Through simulations, phantom experiments, and in vivo application in 20 patients, we demonstrated the technical feasibility of acquiring spatially resolved relaxometry maps.

## Funding

This work was supported by the Dutch Top Sector Life Sciences & Health (Health˜Holland) through the TKI‐LSH grant scheme, as part of the project “Development of MRI based endovasculAR procedures for Vascular surgerY (MARVY).” We gratefully acknowledge this funding support.

## Supporting information


**Figure S1:** Comparison of SPGR and I‐SPGR signals under varying RF spoiling conditions. (a) Simulated signal evolution at a low flip angle (5°) shows almost no deviations from the signal with perfect spoiling. (b) At a higher flip angle (15°), imperfect spoiling leads to more pronounced deviations, both for the SPGR and I‐SPGR signals. (c) Signal variation across RF phase increments at a flip angle of 5° shows I‐SPGR to be robust, while SPGR exhibits irregular sensitivity to the spoiling angle. (d) At a flip angle of 15°, both sequences show increased signal deviations, with I‐SPGR displaying slightly less dependencies on the spoiling angle. All simulations are run with TR = 5 ms, *T*_*FS*_ = 100 ms, *T*_*BS*_ = 12 ms, and *T*_*Sp*_ = 10 ms and isochromats with T1: 500 ms and T2: 20 ms.
**Figure S2:** Fitting errors for the I‐SPGR signal model under simulated noise conditions (SNR = 30). Unlike SPGR, I‐SPGR exhibits substantial errors in T1, T2, and M0 estimation—particularly at low ground‐truth T2 values (< 40 ms), where T1 and M0 errors can exceed 60%. T2 estimation is also unreliable when T1 is below ∼1000 ms, indicating greater sensitivity of I‐SPGR to noise.
**Figure S3:** T1 and M0 fitting errors for the SPGR signal model under simulated noise conditions. With additive Gaussian noise (SNR = 30), SPGR maintains accurate estimation of T1 and M0 across a range of T1 and M0 values, showing only minor fitting errors. These results demonstrate SPGR's robustness to moderate noise.
**Figure S4:** Relative fitting errors for T1, T2, and M0 using optimized acquisition parameters for the I‐SPGR sequence. Top row: The T1‐optimized configuration (TR = 19 ms, *T*_*FS*_ = 20 ms) substantially reduced fitting errors in T1 and M0, with moderate improvement in T2 estimation—though errors at low T2 values still reached ∼40%. Bottom row: In contrast, optimizing for minimal T2 error (TR = 8 ms, *T*_*FS*_ = 62 ms) yielded the lowest T2 errors, particularly at higher T1 values, while T1 and M0 errors improved modestly compared to the original acquisition but remained higher than in the T1‐optimized case.
**Figure S5:** Same day repeatability (a, c, e) and reproducibility (b, d, f) of T1 and T2 measurements in the phantom for SPGR T1 (a, b), I‐SPGR T1 (c, d), and I‐SPGR T2 (e, f). Bland–Altman plots show the difference between repeated measurements (Measurement 1–Measurement 2) as a function of their mean, along with the mean difference (dashed green line) and the 95% limits of agreement (±1.96 SD; dashed red lines). Within‐subject coefficient of variation (wCV) values are reported for each sequence.
**Figure S6:** Representative I‐SPGR images from one volunteer with psoas muscle and aortic wall indicated (red). Note that to show signal changes over FAs, window‐level was kept the same, resulting in some images being saturated and others being low in intensity.
**Figure S7:** Bland–Altman analysis of T1 (a) and T2 (b) measurements obtained from repeated same‐day healthy volunteer acquisitions using the I‐SPGR sequence. Plots show the difference between measurements (Median T1 or T2 scan 1–Median T1 or T2 scan 2) versus their mean (1/2 × Median T1 or T2 scan 1 + 1/2 × Median T1 or T2 scan 2), along with the mean difference (dashed green line) and 95% limits of agreement (±1.96 SD; dashed red lines). Within‐subject coefficient of variation (wCV) values are reported.

## Data Availability

All simulation code and parameters are provided at https://github.com/wastehling/T1T2‐mapping‐BB‐FS.git.
